# CARDIAC Score: A Framework for a Clinical Tool to Screen and Risk Stratify Pediatric Myocarditis, and Call for Collaboration to Evaluate Use

**DOI:** 10.7759/cureus.105151

**Published:** 2026-03-13

**Authors:** Lauren A Gould

**Affiliations:** 1 Emergency Medicine, Lakeland Regional Hospital, Lakeland, USA

**Keywords:** acute fulminant myocarditis, acute myocarditis in children, endomyocarditis, pediatric clinical cardiology, perimyocarditis, screening strategy, screening tool

## Abstract

There are no clinical decision-making tools to screen for pediatric myocarditis during initial evaluation in the current literature, so we aim to create one using the CARDIAC score. The CARDIAC score is an acronym of its six components: chest X-ray, appearance, risk factors/clinical signs, diagnostics, interventions, abnormal tachycardia, and cardiac markers and C-reactive protein (CRP). Each of these may be scored with 0, 1, or 2 points, with diagnostics and interventions having a third point available to score if the patient meets multiple requirements (i.e., both abnormal electrocardiogram and cardiac point-of-care ultrasound results for diagnostics criteria or requires both respiratory and blood pressure support for interventions required criteria). This is a framework for the proposed CARDIAC score to screen for pediatric myocarditis and risk-stratify patients based on severity and need for further evaluation with echocardiogram, pediatric cardiology consultation, possible cardiac MRI/biopsy, and evaluation for additional interventions such as ECMO (extracorporeal membrane oxygenation) or heart transplantation by a pediatric tertiary or quaternary center. This is a protocol that has not yet been validated and should not be used in clinical practice until validation studies can confirm or reject its utilization.

## Introduction

Pediatric myocarditis is an inflammatory condition of the heart muscle caused by the release and activation of immune cells within cardiac muscle with acute inflammatory properties, including mediators such as tumor necrosis factor-α (TNFα), interleukin-1β (IL-1β), interleukin-6 (IL-6), and nitric oxide [1]. Cardiac fibroblasts respond to injured heart tissue and have also been identified as potent cytokines and chemokine producers, further potentiating the inflammatory process associated with myocarditis [2]. In children, myocarditis is most commonly caused by a viral infection, although other infections, autoimmune diseases, toxins, and drug hypersensitivity can also contribute [1]. The most common viral causes include adenovirus, parvovirus 19, and herpesvirus 6 [1,3,4]. In recent years, COVID-19 and COVID-19 vaccines have been associated with myocarditis primarily in teens and young adults, especially those diagnosed with multisystem inflammatory syndrome in children (MIS-C), which can involve the heart muscle and heart arteries in some infected patients [1].

A history of a viral prodrome preceding myocarditis occurs in about half to two-thirds of pediatric myocarditis patients, and up to 45% present with arrhythmias, including atrial or ventricular arrhythmias or high-grade atrioventricular block [1,5,6]. Some of the most common presenting symptoms for pediatric myocarditis include fatigue (25-70% of patients), shortness of breath (35-69%), fever (31-58%), nausea/vomiting or abdominal pain (28-48%), and chest pain (24-42%) [1]. Some of the most common signs of pediatric myocarditis include tachypnea (52-60% of patients), tachycardia (32-57%), hepatomegaly (21-50%), respiratory distress (21-47%), and cardiac murmur (26%) [1]. Diminished pulses are only present in about 16-21% of patients, and peripheral edema is seen in less than 10% of pediatric patients [1].

Pediatric myocarditis can progress to dilated cardiomyopathy and cardiogenic shock not responsive to medical management, requiring ventricular assist devices or heart transplantation [7]. Post-mortem data identify myocarditis in 8.6% to 12% of cases of sudden death in young adults [8]. Fulminant myocarditis is when the disease has progressed to its most severe form, causing myocardial edema and myocyte necrosis, leading to refractory cardiogenic shock and severe outcomes in nearly 38% of pediatric patients, including death, survived sudden cardiac death, need for a left ventricular assist device, decompensated heart failure, heart transplantation, catecholamine therapy, or malignant arrhythmia [5,9]. Pediatric myocarditis can have catastrophic effects on children, including the need for cardiac transplantation or death, so early recognition and diagnosis are imperative to optimize outcomes with early treatment and supportive care.

Laboratory tests such as cardiac troponin or brain natriuretic peptide (BNP or pro-BNP) levels can be used as markers to assist in screening for myocarditis. Troponin elevation indicates myocardial injury, and BNP elevation indicates cardiac strain and is commonly elevated with left ventricular dilation and dysfunction. In patients with acute myocarditis, studies have demonstrated near-universal troponin elevation amongst these patients with variably elevated or normal BNP levels [10,11]. While troponin elevation is more common in myocarditis, BNP elevation and inflammatory marker (C-reactive protein [CRP]) elevation may be associated with worse prognosis and increased complications [11,12]. In patients with normal troponin, BNP, and CRP laboratory results, the diagnosis of clinically significant myocarditis is very unlikely but cannot be ruled out with certainty without confirmatory imaging or biopsy [13].

There are several frameworks related to myocarditis, including the AHA 2021 Scientific Statement, providing a diagnostic algorithm with four certainty strata ranging from confirmed diagnosis with cardiac biopsy or cardiac MRI to clinically suspected myocarditis without definitive confirmation [1]. Several myocarditis-related scoring systems exist, including the acute myocarditis child death risk score (AMCDRS), which is a 10-variable pediatric mortality prediction model for acute myocarditis [14]; the SAMY score, which distinguishes myocarditis from acute coronary syndrome in adults [15]; and the Lake Louise Criteria, which provides cardiac MRI-based diagnostic criteria with a sensitivity of 87.5% and a specificity of 96.2% [16,17]. While all of these relate to myocarditis, there are no validated screening clinical prediction rules that exist for initial screening of suspected myocarditis upon earliest evaluation in the emergency department.

The CARDIAC score was developed based on clinical experience and medical literature review and designed to be an easy-to-use clinical tool similar to the HEART score for adult chest pain patients and the APGAR score for newborns. The CARDIAC score is an acronym of its six components: chest X-ray, appearance, risk factors/clinical signs, diagnostics, interventions, abnormal tachycardia, and cardiac markers and CRP. Each of these may be scored with 0, 1, or 2 points, with diagnostics and interventions having a third point available to score if the patient meets multiple requirements. For example, both abnormal electrocardiogram (ECG) and cardiac point-of-care ultrasound (POCUS) results are diagnostic criteria, or both respiratory and blood pressure support are required for intervention criteria. Most of the components for the CARDIAC score are objectively determined, except for Appearance, which uses subjective patient history and physician judgment, although some objective physical signs can be used. Signs of moderate to severe respiratory distress include tachypnea, retractions, nasal flaring, labored breathing, gasping, wheezing/rales, cyanosis, and hypoxia. Signs of shock include delayed capillary refill, poor perfusion, hypotension, pallor, mottling, cyanosis, and weak/absent pulses.

There are no clinical prediction rules to screen for pediatric myocarditis in the current literature, so the author aims to create one using the CARDIAC score and evaluate its use through retrospective and/or prospective studies to disprove or validate its possible use in pediatric patients under 18 years of age to screen for myocarditis. Patients can be risk-stratified for adverse events, including acute or fulminant heart failure/cardiogenic shock, need for extracorporeal membrane oxygenation (ECMO), need for cardiac transplantation, and death. The author planned to perform a retrospective study to evaluate the use of the CARDIAC score, but the institutional data were insufficient to provide an adequate number of patients. This is a protocol that has not yet been validated and should not be used in clinical practice until validation studies can confirm or reject its utilization. The author would like to invite others from children’s hospitals and pediatric centers to collaborate on a project to evaluate the use of the CARDIAC score.

## Materials and methods

This is a framework for the proposed CARDIAC score to screen for pediatric myocarditis and risk-stratify patients based on severity and need for further evaluation with an echocardiogram, pediatric cardiology consultation, possible cardiac MRI/biopsy, and evaluation for additional interventions such as ECMO or heart transplantation by a pediatric tertiary or quaternary center. This is a protocol that has not yet been validated and should not be used in clinical practice until validation studies can confirm or reject its utilization.

Patient population and data collection

Study population inclusion criteria will include pediatric patients under the age of 18 with a diagnosis upon admission or discharge of myocarditis, myopericarditis, myoendocarditis, shortness of breath, chest pain, tachycardia, elevated troponin, elevated pro-BNP, respiratory distress, cardiogenic shock, sepsis, septic shock, or heart failure. Exclusion criteria will include those with missing data on any of the criteria assessed within the preliminary CARDIAC score, as shown in Table 1. The proposed thresholds are subject to data-driven calibration based on results from validation studies. The Clinical Informatics department will provide the investigators with a list of encounter identification numbers based upon the patient population within the electronic data warehouse that meets the above inclusion and exclusion criteria. The investigators will then manually review each case for the collection of the research data elements listed below. Chart review will occur by two independent physicians, and any discrepancies between the two-chart reviewing processes will result in a third physician reviewing and making the final decision. Institutional review board (IRB) approval was obtained with a waiver of informed consent on September 18, 2025, with IRB number FY2025-47.

CARDIAC score development

The CARDIAC score shown in Table 1 was derived from a literature review and clinical experience. Components of the CARDIAC score, including chest X-ray, diagnostics, and interventions, were determined based on other peer-reviewed publications that assist in clinical diagnosis, prognostication, and illness severity of patients with myocarditis [1,5-9,14]. Components, including appearance, risk factors, clinical signs, and abnormal tachycardia, were developed based on literature describing common prodromes and presenting symptoms of patients with confirmed myocarditis diagnoses [1,3,4] and pediatric advanced life support (PALS) normal pediatric vital signs criteria [18]. The final component of cardiac markers and CRP, which includes troponin, BNP, and CRP laboratory blood levels, was determined based on a literature review of peer-reviewed studies that demonstrated near-universal rises in troponin and CRP levels in patients with myocarditis with variable elevations of BNP, although elevated BNP is associated with worse illness and prognosis [10-13].

**Table 1:** CARDIAC score for pediatric myocarditis Risk factors include recent infection, autoimmune conditions, Kawasaki disease, being immunocompromised, and recent COVID-19 vaccination. Clinical signs include the following: hepatomegaly, splenomegaly, and peripheral edema, including bilateral lower extremities edema, cardiac murmur Abnormal cardiac POCUS includes reduced ejection fraction, ventricular dilation or dysfunction, thickened myocardium, and pericardial effusion. *Note: Cardiac pro-BNP markers are elevated at birth and the first several weeks of life in an asymptomatic neonate, which is normal; however, additional signs of respiratory distress, poor perfusion, or heart failure may indicate myocarditis. ECG: electrocardiogram; POCUS: point-of-care ultrasound; CRP: C-reactive protein; MRI: magnetic resonance imaging.

Component	Scoring
Chest X-ray	Normal (0) Cardiomegaly or pulmonary edema (+1) Cardiomegaly AND pulmonary edema (+2)
Appearance	Normal clinical exam (0) Lethargic, poor feeding, fatigue, chest pain (+1) Moderate/severe respiratory distress or signs of shock (+2)
Risk Factors and Clinical Signs*	None (0) 1 risk factor/clinical sign present (+1) 2+ risk factors/clinical signs present (+2)
Diagnostics (ECG and/or cardiac POCUS**)	Normal ECG and cardiac POCUS (0) ST-T changes, arrhythmias, or AV block present on ECG (+1) Abnormal cardiac POCUS (+2) Abnormalities present on ECG and cardiac POCUS (+3)
Interventions Required	None required (0) Requires non-invasive supplemental oxygen (+1) Requires non-invasive positive airway pressure (NIPAP), intubation, or vasopressors (+2) Requires NIPAP/intubation AND vasopressors (+3)
Abnormal Tachycardia	Ages 0-1 year: Heart rate <160 bpm (0) Heart rate sustained >160 bpm (1) Heart rate sustained >180 bpm (2) Ages 1-3 years: Heart rate <120 bpm (0) Heart rate sustained >120 bpm (+1) Heart rate sustained >140 bpm (+2) Age >3 years: No tachycardia (0) Heart rate sustained >100 bpm (1) Heart rate sustained >120 bpm (2)
Cardiac markers and CRP	Normal (0) Elevated troponin > normal (+1) Elevated BNP > normal (for age <1 month use cutoff >1,000 pg/mL) (+1) Elevated CRP > normal (+1) 2 or more elevated above parameters (+2)
Low Risk: 0-4 points (likely alternate diagnosis, monitor for changes in clinical status if moderate/high clinical suspicion) Moderate Risk: 5-9 points (suggest further observation and possible evaluation with echocardiogram and/or cardiac MRI especially if cardiac markers are positive) High Risk: 10-16 points (suggest STAT echocardiogram and/or cardiac MRI, consult cardiology, consider transfer to tertiary center)	

Myocarditis definition and control group identification

Myocarditis patients will be identified using the International Classification of Diseases, Tenth Revision (ICD-10). Myocarditis codings utilized will include ICD-10 codings within I40 (myocarditis), I30.9 (myopericarditis), and I33.9 (myoendocarditis). The control group will be identified using patient diagnoses of shortness of breath, chest pain, tachycardia, elevated troponin, elevated pro-BNP, respiratory distress, cardiogenic shock, sepsis, septic shock, or heart failure who have had blood laboratory studies performed.

Primary objective

To retrospectively apply and evaluate the CARDIAC score in pediatric patients under 18 years of age to screen for myocarditis.

Secondary objectives

Secondary endpoints are the definitive diagnosis of acute myocarditis confirmed with cardiac MRI or biopsy; heart failure/cardiogenic shock; need for ECMO (extracorporeal membrane oxygenation); need for cardiac transplantation; and death.

Proposed risk stratification pathway

**Figure 1:**
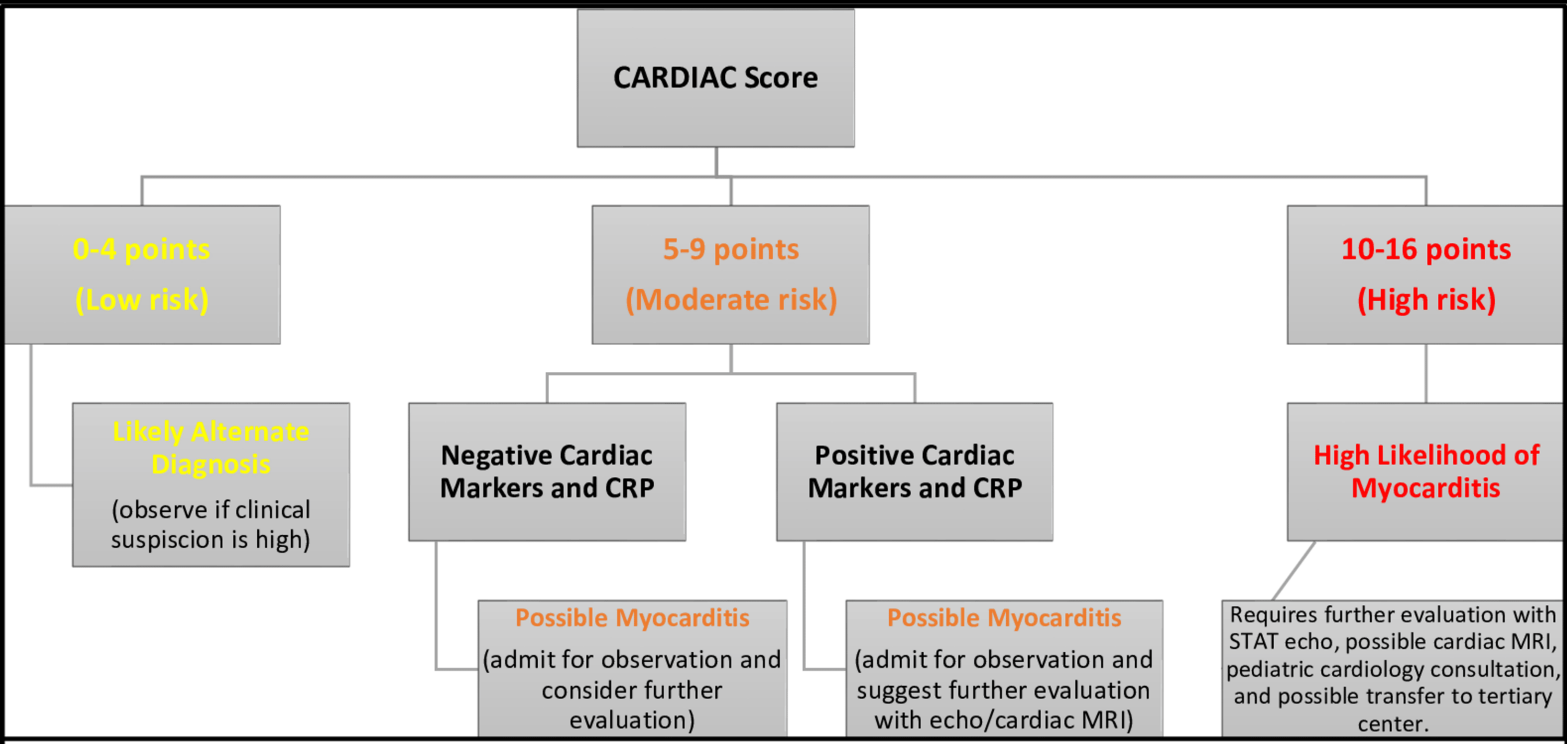
CARDIAC score risk stratification for pediatric myocarditis pathway Schematic of pathway with suggested risk stratification based on CARDIAC score point system and suggested actions to be taken. The proposed thresholds may require revisions following validation studies. CRP: C-reactive protein, MRI: magnetic resonance imaging

A proposed risk stratification pathway based on the CARDIAC scoring is shown as a schematic in Figure 1. A low-risk score of four or less was chosen due to the components of appearance and risk factors/clinical signs having a maximum combined score of four. These components are non-specific and can be seen in a variety of pathologies, including those unrelated to cardiac etiologies, specifically myocarditis. A moderate to severe score over five would indicate additional concerning clinical diagnostic results (i.e., abnormal chest X-ray, ECG, bedside echocardiogram, or laboratory cardiac markers/CRP) or the patient is requiring more aggressive interventions such as respiratory or cardiovascular support. A score over five would indicate the need for further observation at a minimum and likely require additional evaluation with a formal echocardiogram, pediatric cardiology consultation, possible cardiac MRI/biopsy, and evaluation for additional interventions such as ECMO or heart transplantation by a pediatric tertiary or quaternary center. The proposed thresholds are subject to data-driven calibration based on results from future validation studies.

Statistical analysis

All analyses will be conducted within IBM Corp. Released 2024. IBM SPSS Statistics for Windows, Version 30. Armonk, NY: IBM Corp. First, descriptive statistics will be conducted amongst the sampled variables of interest, including means, standard deviations, medians, interquartile ranges, frequencies, and proportions.

Following that, to investigate the primary objective of the present study, we will conduct a hierarchical binary logistic regression modeling procedure, where variables are entered into the model one at a time and evaluated for the overall improvement in model fit indices, including the Akaike Information Criterion, Nagelkerke’s R², and Hosmer-Lemeshow’s goodness of fit test. The area under the receiver operator curve (AUC) will be evaluated and compared across models, additionally to identify the model demonstrating the greatest sensitivity and specificity of the CARDIAC score in predicting the outcome. Individual components of the CARDIAC score will remain in the final model in the event that they significantly improve overall model fit or are statistically significant individual predictors of the outcome. If a variable does not perform either of the aforementioned functions, it will not be included within the final instrument.

Once the optimal CARDIAC score has been determined based on the hierarchical model building procedure, the CARDIAC score category of each case will be utilized when employing chi-square analyses to determine if significant associations between the CARDIAC score category assigned and secondary outcome incidence are observed. Prior to interpretations, assumptions of logistic regression and chi-square analyses will be observed (i.e., linearity of logit, absence of multicollinearity or complete separation, independence, and expected cell counts).

## Results

This is a protocol and call for collaboration to be published with the intention of evaluating the use of the CARDIAC score for pediatric myocarditis.

## Discussion

There are no clinical decision-making tools to screen for pediatric myocarditis in the current literature, so the author aims to create one using the CARDIAC score and evaluate its use through an initial retrospective study using data obtained through collaboration with larger pediatric institutions and children's hospitals.

**Figure 2:**
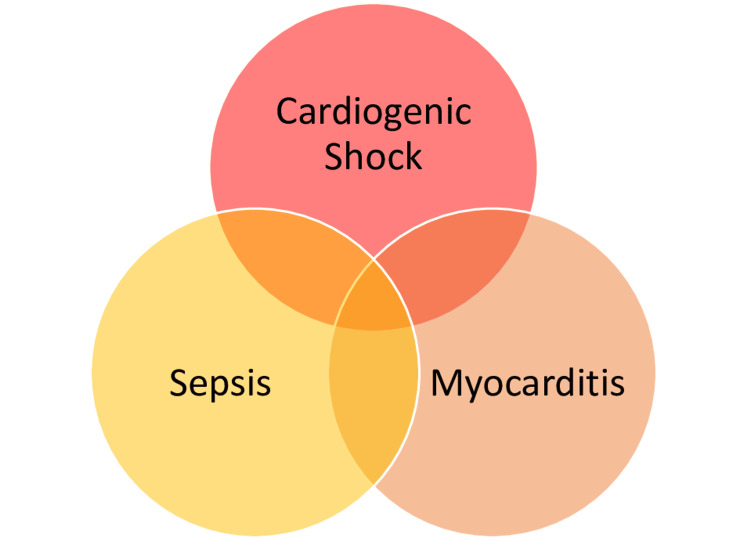
Relationship between sepsis, cardiogenic shock, and myocarditis The differentiation and relationship between sepsis, cardiogenic shock, and myocarditis are important. Sepsis is a life-threatening organ dysfunction caused by an exaggerated immune response by the host to infection. Sepsis can cause cardiomyopathy and may overlap with myocarditis, especially in viral or bacterial infections that directly involve the heart. Myocarditis is inflammation of the heart muscle due to infection, autoimmune responses, or toxins causing myocardial dysfunction and impaired cardiac contractility. Cardiogenic shock is inadequate tissue perfusion due to primary cardiac failure and can be caused by severe myocarditis or sepsis-induced myocardial dysfunction, as well as other primary causes such as myocardial infarction.

It is important to emphasize that the CARDIAC score is meant to be a screening and risk stratification tool for pediatric myocarditis to improve long-term outcomes by expediting transfer to tertiary/quaternary centers for definitive care if patients are moderate- to high-risk. It should be noted that the derivation data were not used, and this is a limitation of the scoring system. The CARDIAC score is not meant to be a diagnostic tool because a cardiac biopsy or cardiac MRI is required for definitive diagnosis. Myocarditis overlaps with other causes of cardiac dysfunction, including sepsis and cardiogenic shock, as shown in Figure 2. If myocarditis is suspected, then the CARDIAC score in theory would be applied to screen and risk stratify patients and identify those that are moderate to high risk and require further evaluation and treatment with a higher level of care at a pediatric tertiary/quaternary center for further testing and definitive care. It is important to emphasize that the CARDIAC score has not been validated and therefore should not be used in clinical practice before appropriate validation studies can be completed.

## Conclusions

Due to limited myocarditis cases available at the author’s current institution, a retrospective analysis was unable to be performed with an adequately powered study to evaluate the utilization of the CARDIAC score. The author would like to invite clinicians from other institutions, specifically children's hospitals and other pediatric institutions, to collaborate to initially retrospectively evaluate the use of the CARDIAC score using the methods described above. If retrospective analysis supports the use of the CARDIAC score in identifying myocarditis and risk-stratifying patients, then prospective validation of the CARDIAC score will be pursued.
